# Microsurgery for intracranial aneurysms: A qualitative survey on technical challenges and technological solutions

**DOI:** 10.3389/fsurg.2022.957450

**Published:** 2022-08-04

**Authors:** W. R. Muirhead, H. Layard Horsfall, D. Z. Khan, C. Koh, P. J. Grover, A. K. Toma, P. Castanho, D. Stoyanov, H. J. Marcus, M. Murphy

**Affiliations:** ^1^ Department of Neurosurgery, The National Hospital for Neurology and Neurosurgery, London, United Kingdom; ^2^ The Wellcome Centre for Interventional and Surgical Sciences, University College London, London, United Kingdom

**Keywords:** aneurysm clipping, subarachnoid haemorrhage, technology, survey, microsurgery

## Abstract

**Introduction:**

Microsurgery for the clipping of intracranial aneurysms remains a technically challenging and high-risk area of neurosurgery. We aimed to describe the technical challenges of aneurysm surgery, and the scope for technological innovations to overcome these barriers from the perspective of practising neurovascular surgeons.

**Materials and Methods:**

Consultant neurovascular surgeons and members of the British Neurovascular Group (BNVG) were electronically invited to participate in an online survey regarding surgery for both ruptured and unruptured aneurysms. The free text survey asked three questions: what do they consider to be the principal technical barriers to aneurysm clipping? What technological advances have previously contributed to improving the safety and efficacy of aneurysm clipping? What technological advances do they anticipate improving the safety and efficacy of aneurysm clipping in the future? A qualitative synthesis of responses was performed using multi-rater emergent thematic analysis.

**Results:**

The most significant reported historical advances in aneurysm surgery fell into five themes: (1) optimising clip placement, (2) minimising brain retraction, (3) tissue handling, (4) visualisation and orientation, and (5) management of intraoperative rupture. The most frequently reported innovation by far was indocyanine green angiography (84% of respondents). The three most commonly cited future advances were hybrid surgical and endovascular techniques, advances in intraoperative imaging, and patient-specific simulation and planning.

**Conclusions:**

While some surgeons perceive that the rate of innovation in aneurysm clipping has been dwarfed in recent years by endovascular techniques, surgeons surveyed highlighted a broad range of future technologies that have the potential to continue to improve the safety of aneurysm surgery in the future.

## Introduction

Victor Horsley surgically treated an intracranial aneurysm with ligation of the internal carotid artery in 1885 (1, 2), and Walter Dandy performed the first clip ligation of an intracranial aneurysm in 1937 (3). In the 1960s and 1970s, Yasargil introduced the modern microsurgical techniques of dissection through cisternal corridors (4–6). Neurosurgical luminaries have further contributed to the field with advances in hypothermic cardiac standstill (7), basilar apex techniques (8–10), bypass (11–13) and skull base approaches (14, 15), expanding the range of aneurysms that could be effectively treated surgically. With the advent of the endovascular era, there has been a decline in the proportion of aneurysms undergoing surgical clipping but also an opportunity for treatment using hybrid open and endovascular and open techniques. Endovascular treatment, which started with simple coiling introduced by Gugliemi in 1990, became increasingly popular after the ISAT trial results in 2002, which demonstrated better outcomes for patients with ruptured aneurysms, which were felt to be suitable for either surgical clipping or simple coiling (16, 17). The decision of whether a patient should be treated with clipping or coiling has been further informed by the BRAT trial (18, 19) as well as further non-randomized analyses typically performed on specific patient subgroups (20, 21). Even with modern endovascular and open bypass techniques, there remains a subset of aneurysm patients that are exceptionally challenging to treat (22, 23). The rate of progress of techniques driven both by innovative practitioners and new technologies from device companies has rapidly increased the number of aneurysms for which there is an endovascular treatment option. Jacques Moret introduced balloon-assisted coiling in the early 1990s, and this was followed by stent-assisted coiling, double microcatheter techniques, flow-diverting stents, and most recently intrasaccular flow diverters starting with the Woven Endoluminal Bridge device (17). During this period of very rapid development in endovascular techniques, there have been relatively fewer changes to clipping techniques with many aneurysms clipped today using techniques very similar to those in Yasargil's description 50 years ago. This is not to say that newer technologies such as indocyanine green angiography (24), microdoppler (25), bypass (13, 26), and hybrid techniques have not flourished with real benefit for patients but simply that the apparent rate of innovation has been slower in the open versus endovascular repair (1, 2).

Despite the long history of innovation in open aneurysm repair, treatment remains technically demanding with a high complication rate (28). In one series of ruptured aneurysms treated with surgical clipping, 36% of the deaths and permanent disabilities were attributed to technical intraoperative complications (29). The profile of adverse events is, of course, recognised to be very different for surgery for unruptured cerebral aneurysms, which have much lower morbidity, principally due to the better condition of the brain and dramatically lower risk of intraoperative rupture (30). There is, of course, extensive literature from the leading cerebrovascular surgeons in the field describing the techniques and challenges of aneurysm surgery including advanced techniques that push the envelope of what is possible (1, 31–36). The aim of this study was to survey a cohort of neurovascular surgeons to describe what they perceive as the principal technical challenges of aneurysm surgery, the technological advances that have been most significant, and how they expect technology to advance the field in the future.

## Materials and methods

A qualitative study design was adopted to allow for a broad range of responses to the perceived technical challenges and solutions of aneurysm surgery. Saturation of data collection in qualitative surveys is said to occur when no significant new information emerges with future participants. In social science research, where subjects are interviewed, this is typically ensured by reassessing for saturation during the data collection process. In our study, where we proposed to capture all the data and only perform analysis *post hoc*, we were keen to ensure that we would have sufficient participants that saturation would have occurred. We estimated this would require approximately 20 participants based on similar studies in other surgical fields (37, 38).

### Participants

Members of the British Neurovascular Group (BNVG) and consultant neurovascular surgeons were electronically invited to participate in an online survey on intracranial aneurysm clipping. A total of 70 BNVG members were invited to participate through an email invitation; additionally, several consultant neurovascular surgeons who were not members of BNVG but known by the research team were approached directly.^1^

### Survey

Respondents were asked to comment on their personal experience with aneurysm clipping, quantified in terms of cases operated, as well as disclose their name and neurosurgical unit to facilitate keeping track of the responses. The open-ended survey asked three questions:

1. What are the principal technical challenges in aneurysm clipping?
2. What technological advances have most contributed to improving the safety and efficacy of aneurysm clipping?
3. What technological advances do you anticipate will improve the safety and efficacy of aneurysm clipping in the future?

No requirement was made to comment on ruptured or unruptured cases separately to allow respondents to cover issues pertinent to both or to focus on one or the other in their responses as they preferred.

### Data analysis

Survey responses were analysed separately by two authors (WM + HLH) to identify the themes. One was the neurovascular fellow, and the other a postgraduate third-year neurosurgical trainee. The author group of this paper further includes four consultant neurovascular surgeons to support the junior authors in the analysis and review the results. Inductive thematic analysis was performed according to previously described methods (39). Responses were parsed for codes that were then amalgamated into subthemes. Once the subthemes were agreed upon by the two authors, the responses were re-examined for expression of the subthemes. Totals were tallied and expressed as percentages. Subthemes were grouped into higher-level themes for the presentation of the results.

## Results

### Participants

**Figure 1:**
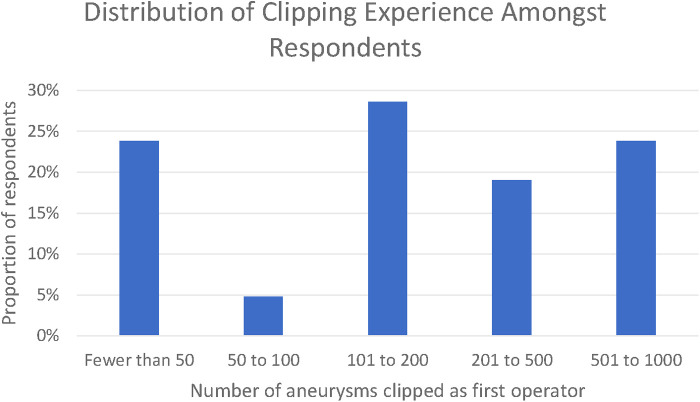
Distribution of clipping experience amongst respondents.

Twenty-one responses were received, whilst the survey was open between 23 January 2021 and the 1 April 2021. Thematic saturation of the qualitative responses was reached at 10, 13, and 18 responses for each of the three open questions, respectively. In total, 76% of respondents had clipped >50 aneurysms and 23% had clipped between 500 and 1,000 aneurysms. None of the respondents had clipped in excess of 1,000 aneurysms (Figure 1).

#### Themes identified in relation to the principal technical challenges in aneurysm clipping

**Figure 2:**
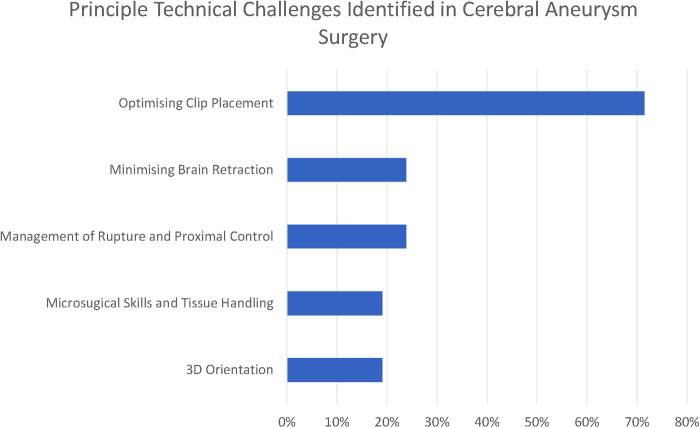
5 Principal technical challenges identified in cerebral aneurysm surgery.

**Figure 3:**
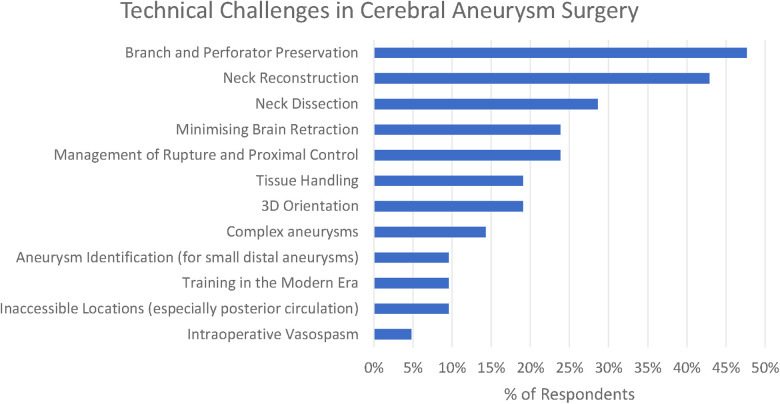
Themes and subthemes identified in technical challenges in cerebral aneurysm surgery (here, the theme of “optimising clip placement” is presented in its three subthemes).

There were five common (reported by at least 15% of respondents) themes that we identified in relation to the principal technical challenges in aneurysm clipping (see Figure 1–3).

Optimising clip placement could be further broken down into three subthemes: Branch and Perforator Preservation, Neck Reconstruction, and Neck Dissection. These are presented along with less frequently reported themes (<15% of respondents) in Figure 2.

The commonest three technical challenges reported by the respondents related to recognising the anatomy of the aneurysm and optimising clip placement are as follows: Branch and Perforator Preservation (48%), Neck Reconstruction (43%), and Neck Dissection (29%).

Perforator presentation was recognised to be a particular challenge in the posterior circulation “For posterior circulation aneurysm (basilar aneurysm, SCA aneurysm, although small group), the challenge is on the perforator”s dissection, preservation and clips occlusion without compromising the perforating vessels” (participant 2), whilst branch preservation is recognised to be in tension with optimal neck reconstruction—“preserving patency of the distal vessels without kinking them. judging how close to the aneurysm neck a clip can be placed without causing complications by aiming for perfection” (participant 11).

In total, 19% of respondents also highlight the challenge of 3D orientation—“understanding of the 3-D local anatomy” (participant 11), “appreciation of 3D anatomy in real time taking into account the brain relaxation and position” (participant 6)—which is particularly relevant in the final stages of the operation when the surgeon is trying to define the anatomy of aneurysm to facilitate safe clip placement.

Minimising brain retraction was identified by 24% of respondents, with participant 1 highlighting that it is difficult to know where many “potentially low-grade symptoms are attributable to even mild retraction”, and this is recognised to be achieved by “skull base surgical principles” (participant 7).

One respondent highlighted the problem of “blood vessel irritability,” leading to intraoperative vasospasm; this went hand in hand with the other surgical principle of good tissue handling including “respect for normal anatomy” (participant 6).

Intraoperative rupture was highlighted by 24%, along with the associated difficulty of “proximal control without compromising the blood flow in the normal brain” (participant 5). A number of respondents identified problems with brain retraction in the context of swollen brain—for numerical purposes there were aggregated under the thematic heading of “minimising brain retraction”. Intraoperative vasospasm was highlighted by a single author (participant 10).

Three challenges relating to specific aneurysm morphologies were highlighted by the responses: large aneurysms (14%), aneurysms in inaccessible locations (10%), and identification of small distal aneurysms (5%). For large and giant aneurysms, a particular concern was “calcified necks” (participant 1). “The deep narrow corridor to get to posterior circulation aneurysms is another challenge” (participant 2). Participant 2 highlighted that whilst identification of small distal aneurysms had previously been difficult, this was now significantly helped by neuronavigation.

The difficulty of training new surgeons and, in particular, of “learning aneurysm surgery in the current era” (participant 6) with increasing intolerance of adverse events was recognised by 10% of participants.

#### Themes identified in relation to historical advances in aneurysm clipping

**Figure 4:**
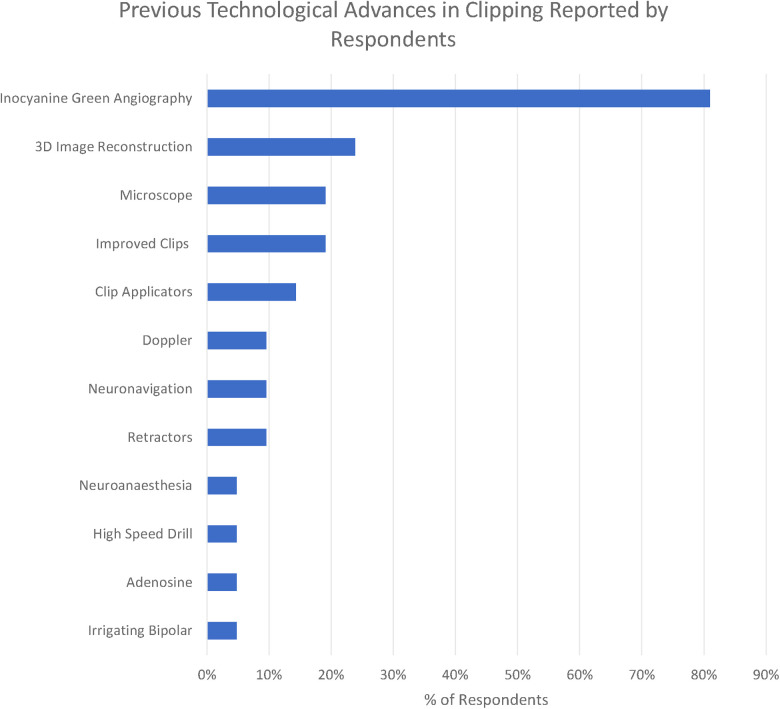
Previous technological advances in clipping reported by respondents.

We identified 11 technologies (treated as subthemes for the purpose of the qualitative analysis) in response to the question “What technological advances have most contributed to improving the safety and efficacy of aneurysm clipping? (Figure 4)”

The five principal operative challenges identified in the previous question informed our thematic grouping, and we considered each of the technological modalities as a subtheme grouped by the technical challenge it was mostly targeted towards addressing.

The overwhelmingly most cited response was indocyanine green angiography (ICG), reported by 81% of respondents. ICG was recognised to be a major breakthrough in optimising clip placement for its ability to identify kinked distal vessels in particular, but an acknowledged limitation is that it does “not address deep perforators” (participant 2). Doppler was highlighted by 10%, although with the recognition that “access is poor in some locations” (respondent 11). Clip technology both of clips themselves (19%) and clip applicators (14%) was also cited. Rotating applicators, in particular, were highlighted by two participants (6 and 19), while others drew attention to “low profile applicators” (participant 14). For the clips themselves, a wider “clip range and geometry” (participant 10) was highlighted.

3D image reconstruction of preoperative CTA, MRA, and DSA was the second most cited advancement (24%). The operating microscope was highlighted by 19% of respondents and neuronavigation by 10% in the context of “small, more distal aneurysm clippings” (participant 2)

A single respondent (participant 10) highlighted the development of the irrigating bipolar as a key technological development in aneurysm surgery, whilst self-retaining retractors (10%), advances in neuroanaesthesia (5%), and the high-speed drill (5%) were all highlighted by respondents. Adenosine (5%) was the only advance highlighted in the responses specifically targeted towards the management of intraoperative rupture.

#### Themes identified in relation to anticipated advances in aneurysm surgery

We identified 12 subthemes in response to the question “What technological advances do you anticipate will improve the safety and efficacy of aneurysm clipping in the future?” Three respondents offered no suggestions at all “Can”t see one at present” (participant 1), “unsure” (participant 16) and “it is difficult to be certain in surgery as the major technological expansion appears to be in endovascular treatment” (participant 10).

The most popular response was Hybrid Surgical and Endovascular Techniques (29%), e.g., “the use of hybrid OR could help determine preservation of deep perforators with some cases of clipping” (participant 1). Participants emphasised both the use of simple on-table check angiography (participant 7) as well as the ability to combine both endovascular and surgical treatments (participant 19).

Advances in clip applicators (10%) as well as clips themselves (14%) were highlighted by respondents. Participant 20 raised the question, “do you need a massive spring on the back of a clip?” Other participants highlighted improvements in bypass techniques including reference to the Elana (excimer laser-assisted nonocclusive anastomosis) technique (26).

Advances in intraoperative imaging were highlighted by 24% of respondents. Miniaturised Optical Coherence Tomography was suggested by participant 20. Technology of the visualisation of “blind” corners was suggested by participant 4. Another suggestion was for augmented reality to deliver “better 3D intraop imaging” (participant 13).

Preoperative simulation particularly tailored to the patient being operated on was suggested by 14% of respondents. “Patient-specific surgical simulation” was suggested (participant 18) as well as 3D models (participant 17).

Robotics (including soft-stiff robotic systems - participant 20) were suggested by 10% of respondents. Artificial intelligence (AI) assistance including “AI assisted clip selection” (participant 6) were also suggested as potential advances.

## Discussion

### Principal findings

**Figure 5:**
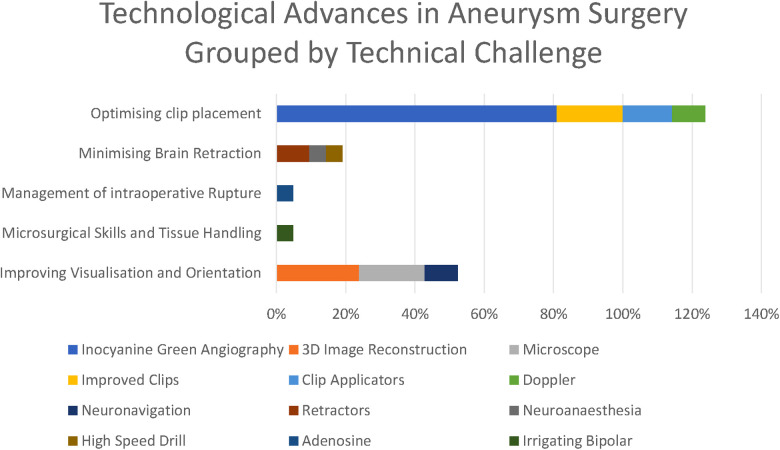
Technological advances grouped by technical challenge they respond to (as percentages express the percentage of respondents citing each technological advance, these percentages do not sum to 100%).

The principle technological advances highlighted in our survey have been driven by five intraoperative challenges: Tissue handling, brain retraction, 3D orientation, optimising clip placement, and the management of rupture and proximal control (Figure 5).

The challenge of training itself was reported by 10% of respondents but deserves special consideration as whilst the other challenges are challenges of specific aspects of the surgery, training a new generation in aneurysm surgery touches on all of the specific technical challenges as all of these must be mastered by future surgeons. Training in this unforgiving technical specialty is recognised to present an increasing challenge in the modern era (40, 41) where we have high expectations of the level of surgery as well as increasingly few opportunities to learn. High-fidelity surgical simulators (42) may offer a partial solution to this, allowing surgeons to gain experience even as the number of aneurysms being clipped decreases.

**Figure 6:**
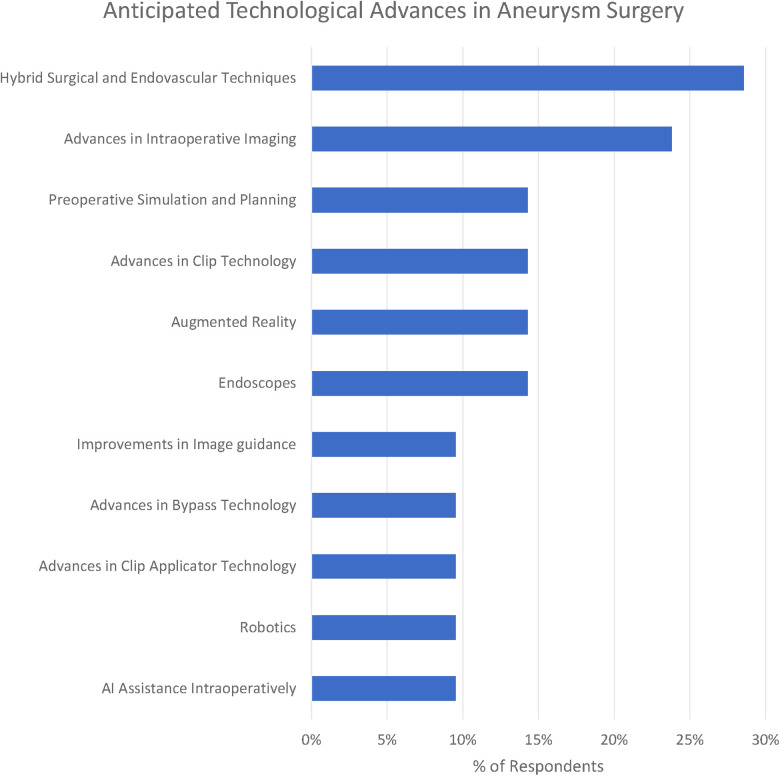
Anticipated technological advances in aneurysm surgery.

The most striking technological advance highlighted by those surveyed is the dominance of ICG (Figure 6) in the responses highlighted by 81% of respondents, making it far and away the most commonly reported technological advance. ICG was first described in 2003 and addressed the core problem of optimising clip placement by allowing visualisation of distal vessels and perforators as well as confirmed exclusion of the aneurysm from the circulation.

When we consider the anticipated advances, the majority of respondents identified technology that would improve clip placement (e.g., advances in applicators, advances in clips) or intraoperative visualisation and orientation (e.g., endoscopes or advances in clip technology). None of the themes identified technologies to improve tissue handling or management of intraoperative rupture despite these being amongst the most frequently cited technical challenges. This highlights potentially under-explored areas for future solutions to target.

Hybrid operating theatres, whilst commonplace in some parts of the world, are a rarity in the United Kingdom, which may explain their inclusion as a future advance here. It may be that the very small number of dual-trained endovascular surgeons in the United Kingdom (compared to, e.g., the United States) is a factor here. Endoscopes, similarly, whilst a longstanding technology, are not typically incorporated into the aneurysm surgical workflow in practice here.

**Figure 7:**
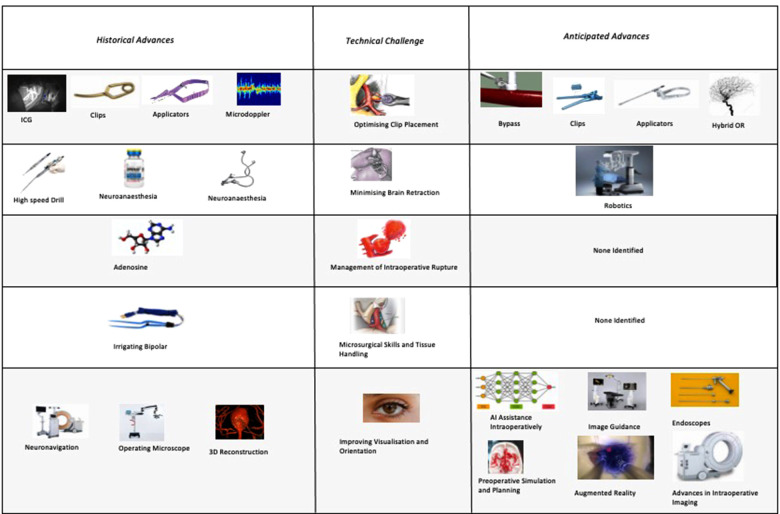
Anticipated advances in cerebral aneurysm surgery grouped by technical challenge (percentages do not sum to 100% as they express the proportion of respondents citing each advance).

The focus of both historical and anticipated advances in aneurysm surgery have focused on optimising clip placement, optimising visualisation, and minimising brain retraction. There has been relatively less technological innovation targeted at improving tissue handling and the management of intraoperative rupture (Figures 7).

### Comparison with other studies

The challenges of minimising brain retraction, anatomical orientation, tissue handling, clip placement, and managing rupture reflect the tenets highlighted by many authorities on aneurysm surgery (27, 11).

Notably, in the literature, the most commonly reported adverse event is intraoperative rupture (28) that may reflect the binary nature of rupture, which lends itself to reporting in case series. The most-reported technical challenge of neck reconstruction/clip placement is reflected in the literature by a rate of suboptimal clip placement being recognised intraoperatively in 15.5% of cases.

Intraoperative monitoring was not identified as a theme in our cohort despite being commonplace in many parts of the world and described by other authors, although the huge contribution made by ICG angiography is certainly reflected in other studies (31, 35).

### Limitations

As our survey was conducted in the United Kingdom it may not totally reflect the views of the global neurosurgery community due to regional variations. In the United Kingdom, the overwhelming majority of aneurysms are clipped by subspecialist neurovascular surgeons but in an environment that has some important differences compared with the large United States centres that feature prominently in the literature. For example, resources are significantly more constrained with 50% lower healthcare expenditure per capita (43), it is much less common for neurovascular surgeons to be dual-trained, and the overall proportion of aneurysms treated endovascularly is higher (44, 45).

Additionally, dual open and endovascular training is rare in the UK, and this may have let to less emphasis on, for example, hybrid theatre suites with on-table angiography than in countries where this is more common.

In response to the very rapid growth of technologically driven advances in endovascular surgery, our survey was focused on the role of technological innovation in aneurysm microsurgery. Consequently, our analysis may underplay the importance of a technically excellent microsurgeon in improving the safety of each case. Respondents did highlight the importance of microsurgical skills and the challenges of training the next generation of neurovascular surgeons. Technology can, of course, improve the apparent skill level of the surgeon either by improving their understanding of the anatomy (46) or providing novel training opportunities (47), but at present, this is at best a partial substitute for the operative experience gained from case volume.

A further factor to consider is that we did not specify the time horizon for either previous or future advances, and this led to a wide variety in the types of responses offered. Whilst the microscope has undoubtedly been revolutionary in the field of aneurysm surgery since it was popularised by Yasargil (1, 2), it seems likely that this is because its use had become widespread by the time many of the respondents began their practice, whereas newer technologies such as ICG have been developed within recent memory.

Due to the focus on technological innovation, it is likely that there was some variation between respondents on what would count as “technology.” Adenosine and neuroanaesthesia were highlighted by some respondents, but it may be that others would have considered them outside the scope of the survey. That there was no mention of either bypass or temporary clipping in the historical technological advances was perhaps for the reason that neither requires specific dedicated technology to enable them.

## Conclusions

Compared with the recent rapid advancement in endovascular techniques there is a perception amongst some surgeons surveyed that the rate of progress in surgical clipping has slowed or may even have stopped completely. The surgical advances highlighted in our survey were directed towards addressing five central problems in aneurysm clipping: optimising clip placement, tissue handling, minimising brain retraction, 3D orientation, and the management of intraoperative rupture. The neurovascular surgeons surveyed highlighted multiple future advances directed towards these problems including simulation, AI and robotics, and advanced visualisation including hybrid open/endovascular techniques.

The majority of future advances identified were targeted at the problems of optimising clip placement (e.g., improved clips and applicators) and visualisation/orientation (e.g., image guidance and endoscopes). There were far fewer future advances identified targeting the problems of brain retraction, tissue handling, and intraoperative rupture, even though these were equally recognised problems in clipping. Future research directed towards these relatively under-addressed challenges may therefore be especially high yield.

The last 30 years have seen rapid advances in endovascular techniques, with patients benefitting from the increased range of treatment options. Whilst microsurgical clipping has not advanced at the same pace, there have still been important developments over this time, perhaps most notably indocyanine green angiography. As endovascular treatments continue to develop, there needs to be a similar focus on technological innovation in open surgical repair so that patients can continue to benefit from whichever treatment modality is best for their aneurysm.

## Data Availability

The datasets presented in this article are not readily available because releasing even an anonymised version of the full dataset may still likely make it possible to identify some participants given the small number of uk neurovascular surgeons. However, we will be happy to share any parts of the data set that is requested providing this partial dataset does not risk making individual respondent identifiable. Requests to access the datasets should be directed to <w.muirhead@ucl.ac.uk>.

## Footnotes

^1^
Membership of the BNVG is not mandatory for consultant neurovascular surgeons. On the basis that there are approximately 30 neurosurgical units in the United Kingdom, each with typically 2–4 neurovascular surgeons, we estimate that there are somewhere between 60 and 120 consultant neurovascular surgeons in the United Kingdom (not all of the BNVG membership are necessarily consultant surgeons).
